# Chronic epididymitis due to *Chlamydia trachomatis* LGV-L2 in an HIV-negative heterosexual patient: a case report

**DOI:** 10.3389/fpubh.2023.1129166

**Published:** 2023-05-09

**Authors:** Daniela Andrea Paira, José Javier Olmedo, Carolina Olivera, Andrea Daniela Tissera, Rosa Isabel Molina, Virginia Elena Rivero, Rubén Darío Motrich, Héctor Alex Saka

**Affiliations:** ^1^Centro de Investigaciones en Bioquímica Clínica e Inmunología (CIBICI), CONICET, Córdoba, Argentina; ^2^Departamento de Bioquímica Clínica, Facultad de Ciencias Químicas, Universidad Nacional de Córdoba, Córdoba, Argentina; ^3^Fundación Urológica Córdoba para la Docencia e Investigación Médica (FUCDIM), Córdoba, Argentina; ^4^Laboratorio de Andrología y Reproducción (LAR), Córdoba, Argentina

**Keywords:** sexually transmitted infections, *Chlamydia trachomatis*, epididymitis, semen, case report

## Abstract

*Chlamydia trachomatis* is an obligate intracellular pathogen and the leading bacterial cause of sexually transmitted infections worldwide. *Chlamydia trachomatis* genovars L1–L3 are responsible for lymphogranuloma venereum (LGV), an invasive sexually transmitted disease endemic in tropical and subtropical regions of Africa, South America, the Caribbean, India and South East Asia. The typical signs and symptoms of *C. trachomatis* LGV urogenital infections in men include herpetiform ulcers, inguinal buboes, and/or lymphadenopathies. Since 2003, endemic cases of proctitis and proctocolitis caused by *C. trachomatis* LGV emerged in Europe, mainly in HIV-positive men who have sex with men (MSM). Scarce data have been reported about unusual clinical presentations of *C. trachomatis* LGV urogenital infections. Herein, we report a case of a 36-year-old heterosexual, HIV-negative male declaring he did not have sex with men or trans women, who presented to the Urology and Andrology outpatient clinic of a healthcare center from Cordoba, Argentina, with intermittent testicular pain over the preceding 6 months. Doppler ultrasound indicated right epididymitis and funiculitis. Out of 17 sexually transmitted infections (STIs) investigated, a positive result was obtained only for *C. trachomatis*. Also, semen analysis revealed oligoasthenozoospermia, reduced sperm viability as well as increased sperm DNA fragmentation and necrosis, together with augmented reactive oxygen species (ROS) levels and the presence of anti-sperm IgG autoantibodies. In this context, doxycycline 100 mg/12 h for 45 days was prescribed. A post-treatment control documented microbiological cure along with resolution of clinical signs and symptoms and improved semen quality. Strikingly, sequencing of the *ompA* gene revealed *C. trachomatis* LGV L2 as the causative uropathogen. Remarkably, the patient did not present the typical signs and symptoms of LGV. Instead, the infection associated with chronic testicular pain, semen inflammation and markedly reduced sperm quality. To our knowledge, this is the first reported evidence of chronic epididymitis due to *C. trachomatis* LGV L2 infection in an HIV-negative heterosexual man. These findings constitute important and valuable information for researchers and practitioners and highlight that *C. trachomatis* LGV-L2 should be considered as putative etiologic agent of chronic epididymitis, even in the absence of the typical LGV signs and symptoms.

## Introduction

*Chlamydia trachomatis* is an obligate intracellular bacterium and the etiologic agent of a range of oculo-genital infections representing a huge burden to public health. Based on the variable domains of the major outer membrane protein (MOMP), this bacterium can be sub-classified into genovars A to L. This classification is epidemiologically relevant since different genovars are associated to specific diseases. Genovars A–C are the causative agents of endemic trachoma, a chronic infection of the eye's conjunctiva and the leading cause of infectious blindness (1–3). Genovars D–K are the most frequent bacterial cause of sexually transmitted infections (STI) worldwide, with urethritis and cervicitis being the main clinical presentations in males and females, respectively (1, 3, 4). Noteworthy, more than 50% of *C. trachomatis* genital infections caused by genovars D–K have been estimated to be asymptomatic and long-lasting, producing repeated cycles of tissue damage and scarring that can ultimately result in pelvic inflammatory disease, ectopic pregnancy and irreversible infertility in women (3, 5, 6). On the other hand, *C. trachomatis* genovars L1, L2, L3, and its subvariants are responsible for lymphogranuloma venereum (LGV), a relatively infrequent and invasive sexually transmitted disease (3, 7). Most LGV cases occur in endemic, tropical and subtropical regions of Africa, South America and the Caribbean, India and South East Asia (8).

The classical manifestations of LGV involve three consecutive stages. The first stage occurs up to a month upon infection and may present with symptoms of urethritis, cervicitis, proctitis or it can even be asymptomatic. This stage implies the formation of a primary lesion at the site of inoculation, usually a papule or small herpetiform ulcer that heals spontaneously in a few days. In the second stage, which develops days to weeks upon the resolution of the primary lesion, the bacteria disseminate via the lymphatics and proliferate within lymph nodes and surrounding tissues close to the inoculation site causing inflammation, lymphadenopathy, systemic symptoms and typically the formation of inguinal buboes that may rupture and spontaneously drain to the outside. If left untreated, LGV may progress to a third stage of the disease characterized by a chronic granulomatous inflammatory process leading to severe and frequently irreversible complications in the genital and anorectal tracts, including fibrosis, stenosis, strictures, lymphedema, elephantiasis, and ulceration of the external genitalia both in men and women (3, 9).

In 2003, endemic cases of LGV proctitis and proctocolitis were reported in Europe, clearly associated to HIV-positive men who have sex with men (MSM) (10, 11). Since then, similar LGV cases have been increasingly reported mainly in the MSM community of metropolitan areas in Europe, the USA, Canada and Australia (12–16). Interestingly, the ongoing epidemics of LGV in developed countries displays particular epidemiologic features, including a strong association to MSM and HIV co-infection, proctitis sometimes mimicking Crohn's disease symptoms as the main clinical finding, a significant proportion of asymptomatic cases likely contributing to LGV transmission and, surprisingly, a markedly increased proportion of anorectal compared to genital infections (17–21).

Herein, we report, to our knowledge, the first case of chronic epididymitis due to *C. trachomatis* LGV-L2 infection in an HIV-negative, heterosexual man without the typical signs and symptoms of LGV.

## Case description

**Figure 1:**
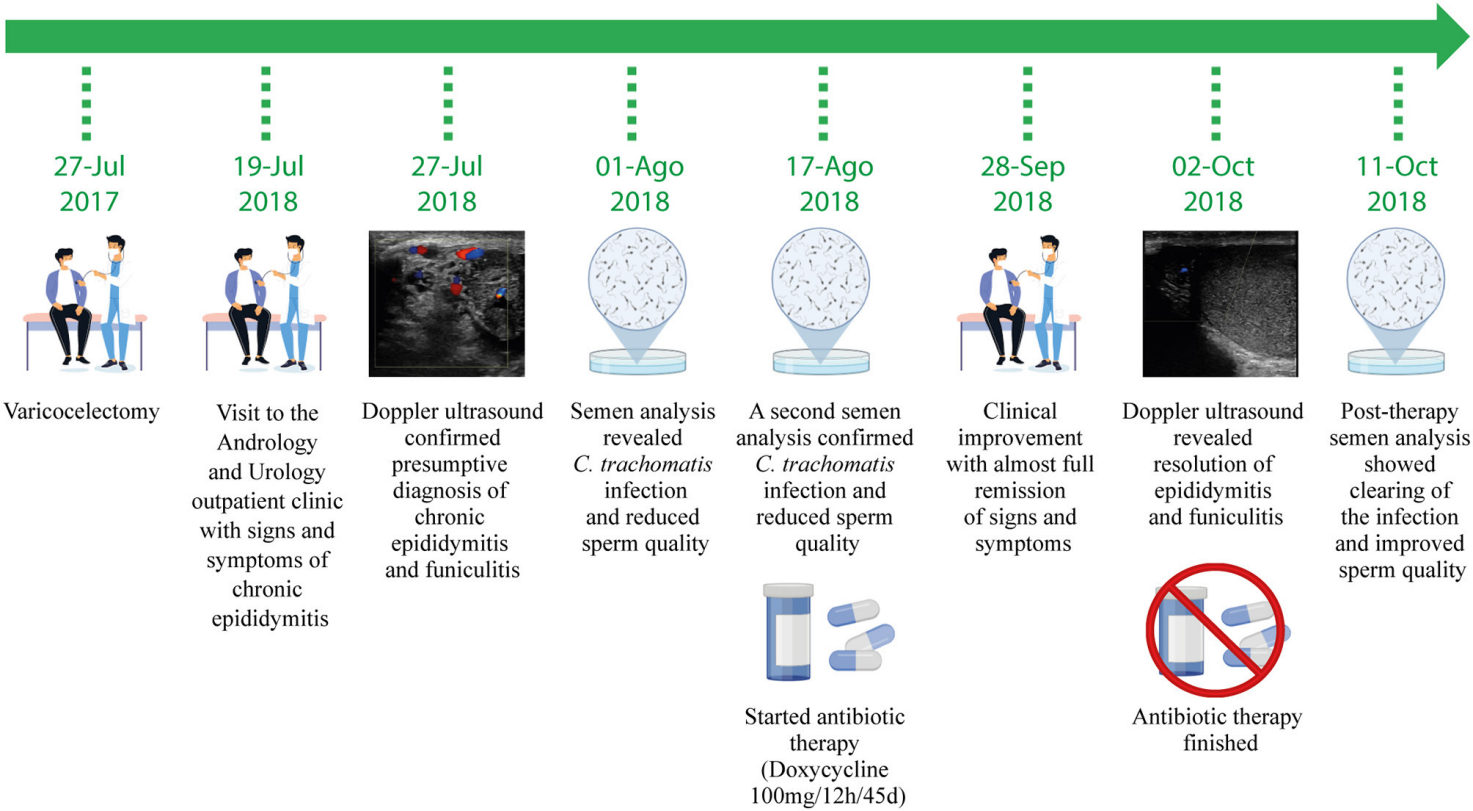
Timeline of the clinical evolution, diagnostic studies, pathogen identification, therapy and outcome of the patient case study.

A 36-year-old male presented to a Urology and Andrology outpatient clinic of a healthcare center from Cordoba, Argentina, in July 2018 referring signs and symptoms of epididymo-orchitis (Figure 1). He is a heterosexual male, married to a woman, declaring not to have had sex with men or trans women, who was also seeking care for couple's primary infertility (attributed to his wife anovulation). The patient complained of intermittent pain and discomfort in the testicles over the last 6 months. His height and weight were 178 cm and 75 kg, respectively, indicating a normal body mass index (BMI: 23.7). Physical examination revealed intense pain on scrotum palpation (particularly in the right epididymis), tender and swollen right epididymis, slightly increased size of the right testicle and enlargement of the right spermatic cord. Blood pressure, heart rate, breathe sounds on auscultation, body temperature and the remainder of the routine physical examinations were normal. The patient's medical record revealed a history of varicocele surgery 1 year earlier (July 2017) (Figure 1). In this context, chronic epididymo-orchitis was considered as a presumptive diagnosis and a series of imaging and laboratory studies were indicated.

## Diagnostic assessment

**Figure 2:**
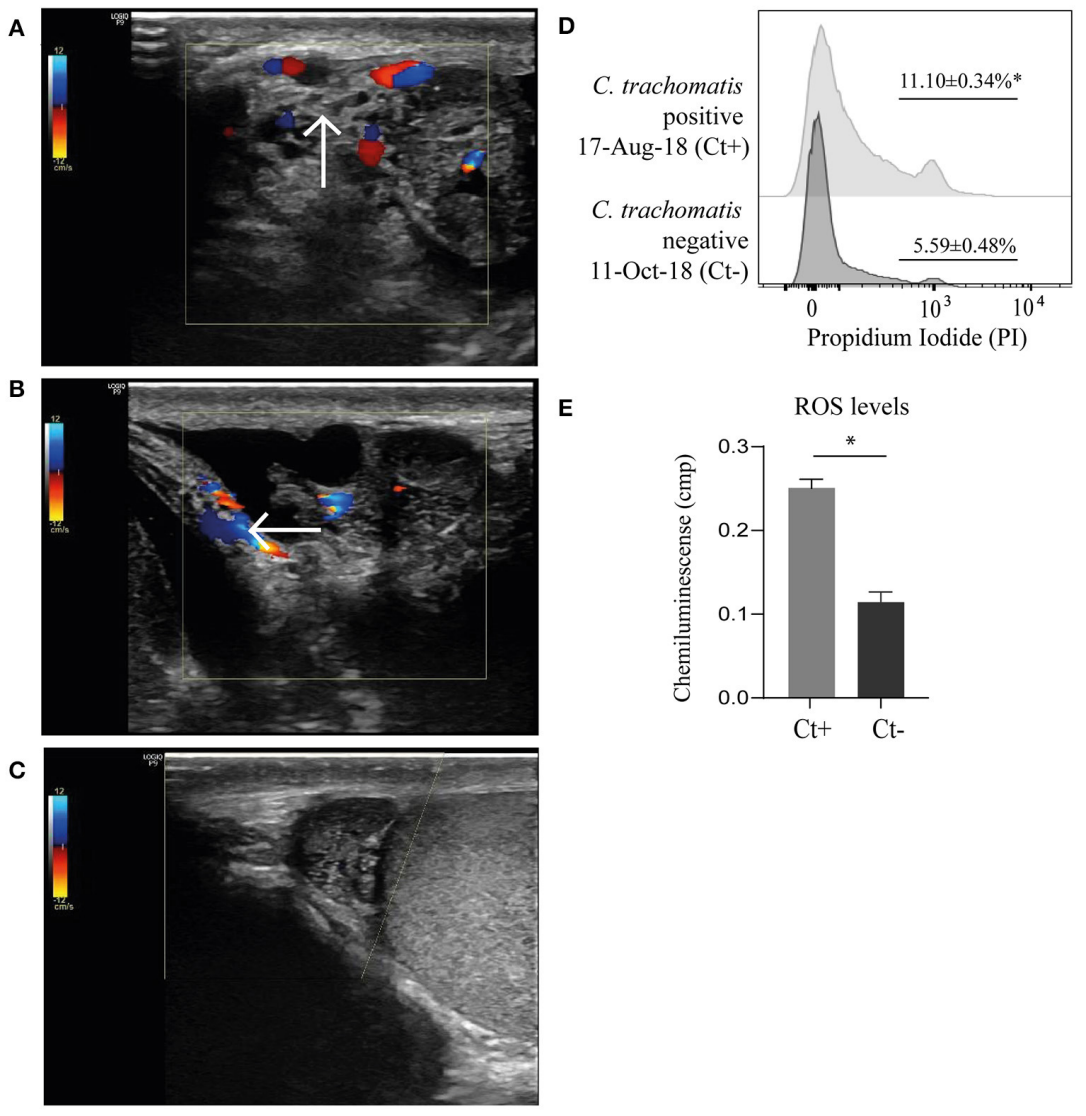
Testicular color Doppler ultrasounds showing hyperechogenicity and enlarged right epididymis with markedly increased vascularity that extended to the fatty spermatic cord compatible with epididymitis **(A)** and funiculitis **(B)** at patient admission (indicated with arrows), and almost complete resolution of tissue changes after antibiotic therapy and infection clearing **(C)**. Assessment of sperm viability by flow cytometry using propidium iodide staining as previously described (22) **(D)**. Representative histograms depicting frequencies of necrotic spermatozoa (propidium iodide-positive) within the spermatozoa population gated on FSC vs. SSC plots. Data were collected on FACS-CANTO II flow cytometer (BD Biosciences, San Diego, USA) and analyzed using FlowJo software (version 7.6.2). Proper compensation using Fluorescence Minus One (FMO) controls were used. Quantification of reactive oxygen species (ROS) level in seminal plasma by chemiluminescence using luminol (Sigma-Aldrich, St. Louis, USA) was carried out according to the WHO laboratory manual for the examination and processing of human semen (23) **(E)**. Measurements were made with use of a Berthold luminometer (model LKB 953; Wallac Inc., Gaithersburg, USA). Results are expressed as percentages (%) or counted photons per minute (cpm). Experiments were performed in triplicates. Data are shown as mean ± SD. Mann–Whitney test; *p<0.05.

A Doppler ultrasound showed hyperechogenicity, augmented size and hypervascularization of the cephalic region of the right epididymis. Moreover, hypervascularization extended to the fatty spermatic cord was observed. These findings were consistent with focal epididymitis and funiculitis (Figures 2A, B). Neither apparent abnormalities nor lesions were observed in the left epididymis and in both testicles.

On August 1st 2018, the patient provided a semen sample (obtained by masturbation) and a panel of 17 sexually transmitted infections (STIs) including Human Immunodeficiency Virus, *C. trachomatis, Ureaplasma urealyticum, Mycoplasma hominis, Mycoplasma genitalium*, Herpes Simplex Virus type 1 and 2, Human Papilloma Virus, *Trichomonas vaginalis, Treponema pallidum, Neisseria gonorrhoeae, Escherichia coli, Pseudomonas* spp., *Streptococcus* spp., *Staphylococcus* spp., *Corynebacterium* spp. and *Candida* spp. were investigated by either PCR or culture as previously described (24). The specific primers used are detailed in Supplementary Table 1. As shown in Table 1, a positive result was obtained for *C. trachomatis* infection while all the other pathogens were negative. The urogenital infection by *C. trachomatis* was later confirmed on August 17th 2018 in an independent sample (Table 1, Supplementary Figure 1, Figure 1).

**Table 1:** Assessment of STI.

**Pathogen**	**Pre-antibiotic therapy**		**Post-antibiotic therapy**
	**01-Aug-18**	**17-Aug-18**	**11-Oct-18**
Human Immunodeficiency Virus^a^	Negative	ND	ND
*Chlamydiatrachomatis* ^b^	**Positive**	**Positive**	Negative
*Ureaplasmaurealyticum* ^b^	Negative	Negative	Negative
*Mycoplasmahominis* ^b^	Negative	Negative	Negative
*Mycoplasmagenitalium* ^b^	ND	Negative	Negative
Herpes Simplex Virus type 1^b^	ND	Negative	Negative
Herpes Simplex Virus type 2^b^	ND	Negative	Negative
Human Papilloma Virus^b^	ND	Negative	Negative
*Trichomonasvaginalis* ^b^	ND	Negative	Negative
*Treponemapallidum* ^b^	ND	Negative	Negative
*Neisseriagonorrhoeae* ^b^	ND	Negative	Negative
*Escherichiacoli* ^c^	Negative	ND	Negative
*Enterococcusfaecalis* ^c^	Negative	ND	Negative
*Pseudomonas* spp.^c^	Negative	ND	Negative
*Streptococcus* spp.^c^	Negative	ND	Negative
*Staphylococcus* spp.^c^	Negative	ND	Negative
*Corynebacteriaceae* ^c^	Negative	ND	Negative
*Candida* spp.^c^	Negative	ND	Negative

Infections were assessed by: ^a^detection of pathogen-specific antibodies (ELISA) or the pathogen nucleic acid (Nucleic Acid Test) in blood samples, ^b^detection of the pathogen by PCR in semen samples, or by ^c^microbiological culture of semen samples as previously described (24). ND, not determined. Positive results are highlighted in bold.

In addition, semen analysis revealed oligoasthenozoospermia (decreased levels of sperm concentration and motility), reduced sperm viability together with increased sperm DNA fragmentation and the presence of anti-sperm IgG autoantibodies (Table 2). All these findings allowed the diagnosis of chronic epididymitis due to *C. trachomatis* infection and a treatment with doxycycline 100 mg/12 h for 45 days was prescribed (Figure 1). In this context, the patient's wife, who was asymptomatic, was immediately prescribed the proper antibiotic treatment as recommended (3, 25).

**Table 2:** Semen quality.

**Semen variable**	***Chlamydia trachomatis*** **positive**		***Chlamydia trachomatis* negative**	**Lower reference limit^a^**
	**01-Aug-18**	**17-Aug-18**	**11-Oct-18**	
Volume (ml)	5.50	6.2	6.1	≥1.5
pH	7.50	7.60	7.60	≥7.2
Sperm concentration ( × 10^6^/ml)	**5.17**	**9.67**	26.67	≥15.00
Sperm total motility (%)	**24**	**19**	**33**	≥40.0
Sperm progressive motility (%)	**12**	**8**	**18**	≥32
Sperm viability (%)	**54**	88	83	≥58
Normal sperm morphology (%)	6	**3**	4	≥4
Peroxidase-positive cells ( × 10^6^/ml)	0.01	0.01	0.06	≤ 1.00
Anti-sperm IgG antibodies (MAR test, %)	**18**	ND	**28**	<10
Sperm chromatin maturity (AB test, %)	84	81	87	≥70
Sperm DNA fragmentation (TUNEL test, %)	**36**	ND	**24**	<20

^a^Lower reference value according to the World Health Organization Semen Analysis Manual 5th Ed. 2010. ND, not determined.

MAR, mixed antiglobulin reaction; AB, aniline blue; TUNEL, terminal deoxynucleotidyl transferase dUTP nick end labeling.

Bold numbers indicate values out of reference range values.

Once the antimicrobial treatment was completed, the patient reported complete resolution of clinical signs and symptoms. A post-treatment Doppler ultrasound showed scarce to absent hypervascularization and slightly increased size and echogenicity of the right epididymitis, findings that were compatible with sequelae of focal chronic epididymitis (Figure 2C). In addition, no signs of inflammation in the right spermatic cord indicated resolution of the funiculitis (Figure 2C). In order to document microbiological cure after treatment, the patient provided a new semen sample on October 11th 2018, which was subjected to infection screening. Negative results were obtained for *C. trachomatis* as well as for all the other pathogens investigated indicating resolution of the infection (Table 1, Supplementary Figure 1, Figure 1). The same results were observed after testing the patient's wife.

Interestingly, a new semen analysis revealed an overall improvement of sperm quality after antimicrobial treatment as shown by increased sperm concentration, viability and motility (Table 2). In addition, levels of reactive oxygen species (ROS) in seminal plasma and necrosis of spermatozoa were evaluated. Moreover, the resolution of the infection associated with significant reductions in ROS and sperm necrosis levels (Figures 2D, E).

In the framework of an ongoing molecular epidemiology study, the identification of the genotype of the *C. trachomatis* strain detected in the patient was performed. For that, sequencing of the MOMP gene (*ompA*) as well as high-resolution genotyping by multilocus sequence typing (MLST) according to the Uppsala scheme were carried out as previously described (26–28). Both, the complete sequence of *ompA* and the MLST allelic profile were successfully obtained. Unexpectedly, the MLST allelic profile showed an exact match with *C. trachomatis* sequence type 141 (ST141). Moreover, the *ompA* gene sequence obtained was 100% identical to that of *C. trachomatis* genovar L2, confirming that the strain detected was *C. trachomatis* LGV L2 (Supplementary Figure 2 and Supplementary Table 2).

Overall, these results indicate that *C. trachomatis* LGV-L2 was identified as the cause of chronic epididymitis in an HIV-negative, heterosexual patient presenting without the typical LGV signs and symptoms.

## Discussion

The epididymis is an important male accessory sex organ where sperm motility and fertilization ability develop. Epididymitis is an inflammation of the epididymis that can be caused by infectious or non-infectious agents and a common cause of male infertility (29). Due to lack of large and comprehensive studies, the exact global prevalence of epididymitis is currently unclear (30). However, it is likely low considering that around 600,000 cases/year occur in the USA (31). Reported data in different settings have shown that epididymitis accounts for 0.69%−1.5% of men presenting to urology outpatient visits (30–32). In line with this, several studies stated a prevalence of epididymitis ranging from 25 to 65 per 10,000 person/year [(33) and references therein].

Although epididymitis can occur in men of any age, the majority of epididymitis cases occur in men aged 20–39 and they are most often associated with STIs (34). In sexually active men younger than 40 years old, *C. trachomatis* and *N. gonorrhoeae* are the most frequent causes of epididymitis (30, 35, 36). However, it is worth mentioning that chlamydial epididymitis usually arises as a complication of ascending urethritis caused by non-LGV *C. trachomatis* genovars (D–K) and mostly presents as an acute episode. In fact, LGV genovars (L1–L3) have not been so far associated to epididymitis (37, 38).

Herein, we report a case of a patient with chronic epididymitis due to *C. trachomatis* LGV-L2 infection associated with oligoasthenozoospermia, necrozoospermia, and increased sperm DNA fragmentation and ROS levels in seminal plasma that significantly improved after antimicrobial treatment and microbiological cure. To the best of our knowledge, this is the first report of *C. trachomatis* LGV-L2 as a causative agent of epididymitis. More unusual, it was in fact a case of chronic epididymitis since signs and symptoms lasted for more than 3 months. Noteworthy, due to its low prevalence, there is very scarce reported data and standardized guidelines for chronic epididymitis clinical management and therapy (32, 39–41).

It is well-known that there is a connection between male infertility and epididymitis/epididymo-orchitis resulting from ascending STIs (42). Inflammatory processes in the epididymis may lead to alterations in sperm count, motility and several sperm functions. In fact, reported evidence indicates that men with epididymitis usually present with impaired semen quality. It has been shown that acute epididymitis associates with reduced sperm concentration, motility and viability (43). The latter could be consequence of pathogen-induced damage and/or inflammation that in turn may result in epididymal dysfunction, tissue scarring and obstruction (29, 44). The drop in sperm concentration observed during the acute phase of epididymitis is usually reversible; however, persistent azoospermia was observed in ~10% of patients and oligozoospermia in another 30% (42, 43). Besides, and in agreement with our data, chronic epididymitis may also result in reduced sperm concentration and motility (45). Interestingly, oligozoospermia has been previously reported during chlamydial epididymitis, whereas no evidence indicates long-term impairment of future fertility (31, 46). Moreover, in most cases of chronic epididymitis, the number of leukocytes in the ejaculate is below the threshold of leukocytospermia in spite of ongoing local inflammation revealed by increased levels of seminal inflammatory cytokines and/or ROS. The latter may in turn impair sperm quality parameters, induce sperm DNA fragmentation and affect several important sperm functions (45). In addition, positive effects of antinflammatory/antibiotic treatment on semen quality have been reported (45). In agreement, it is reasonable to postulate that *C. trachomatis* LGV-L2 chronic infection in the epididymis and the induced local inflammation may be triggering direct or indirect damage to sperm cells, which is supported by the higher levels of ROS in semen, and necrosis and DNA fragmentation in spermatozoa presented by the patient under study prior to antimicrobial treatment and microbiological cure.

Interestingly, the patient also presented anti-sperm IgG autoantibodies. Strikingly, rather than ameliorating, anti-sperm IgG levels increased after antibiotic treatment and microbiological cure of the infection. These results are in line with previous reports pointing out to an association between epididymitis and anti-sperm autoantibodies. Reported data indicates that 27% of patients with acute epididymitis increased the titers of anti-sperm antibodies 3 years after recovery. Moreover, 15% of patients with acute epididymitis developed anti-sperm antibodies *de novo* (43, 47). Physiologically, the epididymal immune balance must be set and maintained toward spermatozoa. The epididymis has a blood-epididymis barrier (BEB) that creates a suitable immune-privileged environment for sperm maturation preventing autoimmune responses against antigenic postpubertal germ cells (48, 49). Infection and inflammation can damage the BEB thus resulting in loss of the immune privilege, which could lead to autoimmunity against sperm antigens (50, 51).

Remarkably, the patient presented with chronic epididymitis but without the typical signs and symptoms of *C. trachomatis* LGV urogenital infections such as inguinal papules or buboes, herpetiform ulcers and/or lymphadenopathy. The causality of the patient's condition related to *C. trachomatis* LGV infection is strongly supported by resolution of clinical signs and symptoms along with microbiological cure after anti-chlamydial antibiotic therapy was implemented. This finding highlights the importance of considering *C. trachomatis* LGV among other common STIs when diagnosing male urogenital infections even in the absence of the typical clinical presentation of LGV. Indeed, atypical clinical presentations of *C. trachomatis* LGV infections have already been reported (14, 52, 53).

As mentioned above, we did not find any previous report of epididymitis caused by *C. trachomatis* LGV genovars pointing out to the novelty of this communication. Interestingly, the detection of this case concurred in time with the re-emergence of endemic cases of urogenital infections caused by *C. trachomatis* LGV in our country after decades (54). Noteworthy, between September 2017 and July 2018, 33 confirmed cases of *C. trachomatis* LGV (mainly L2b genovar) infections were detected in the region. All these infections were detected in MSM, 90% of them HIV-positive, presenting with proctitis as the dominant clinical manifestation (55). Clearly, these emergent cases of *C. trachomatis* LGV infection recently reported in the region share similar epidemiological features with those detected in Europe, Australia and North America, as opposed to the case presented herein.

In summary, this case highlights the importance of considering *C. trachomatis* genovars LGV as a putative cause of epididymitis, even in the absence of the typical LGV signs and symptoms. The findings presented herein delivers important data for researchers as well as clinically useful information for physicians and microbiologists to improve diagnosis and prevent infection transmission and its associated complications. Moreover, our data warrant further molecular epidemiological investigations to elucidate the precise role of *C. trachomatis* LGV genovars in male urogenital infections.

## Patient perspective

The patient has remained stable and with no further evident complications after adhering to the treatment proposed and clearing the infection. He was satisfied with the improvement in the clinical condition and regularly attends to periodic controls.

## Data availability statement

The original contributions presented in this work are included in the article/Supplementary material. Further inquiries can be directed to the corresponding authors.

## Ethics statement

The studies involving human participants were reviewed and approved by Institutional Review Board of the Hospital Nacional de Clínicas, Universidad Nacional de Córdoba (RePIS #3512). The patients/participants provided their written informed consent to participate in this study. Written informed consent to participate in this study was provided by the patient for the publication of any potentially identifiable images or data included in this article.

## Author contributions

RDM and HAS: conceptualization, funding acquisition, resources, supervision, data analysis and curation, writing, and editing of the manuscript. DAP: data acquisition, formal analysis, validation, writing, and editing of the manuscript. JJO: patient care, data acquisition, formal analysis, and validation. CO, ADT, and RIM: methodology and data acquisition. VER: writing and editing of the manuscript. All authors significantly contributed to the article and approved the submitted version.
