# Supplementary material for: Chronic epididymitis due to Chlamydia trachomatis LGV-L2 in an HIV-negative heterosexual patient: a case report
Source: Front Public Health. 2023 May 9;11:1129166. doi: 10.3389/fpubh.2023.1129166 (PMC10203518; doi:10.3389/fpubh.2023.1129166)
Supplement: Supplementary file 1 [file Data_Sheet_1.pdf]

Supplementary Material

Chronic epididymitis due to *Chlamydia trachomatis* LGV-L2 in an HIV-negative heterosexual patient: a case report

Daniela Andrea Paira[1,2], José Javier Olmedo[3], Carolina Olivera[1,2], Andrea Daniela Tissera[4], Rosa Isabel Molina[4], Virginia Elena Rivero[1,2], Rubén Darío Motrich[1,2†*], Héctor Alex Saka[1,2†*].

[1]Centro de Investigaciones en Bioquímica Clínica e Inmunología (CIBICI), CONICET, Córdoba, Argentina.

[2]Departamento de Bioquímica Clínica, Facultad de Ciencias Químicas, Universidad Nacional de Córdoba, Córdoba, Argentina.

[3]Fundación Urológica Córdoba para la Docencia e Investigación Médica (FUCDIM), Córdoba, Argentina.

[4]Laboratorio de Andrología y Reproducción (LAR), Córdoba, Argentina.

[†] These authors contributed equally to this work and share senior authorship.

*** Correspondence:**

Rubén Darío Motrich (rmotrich@unc.edu.ar) and Héctor Alex Saka (alex.saka@unc.edu.ar)

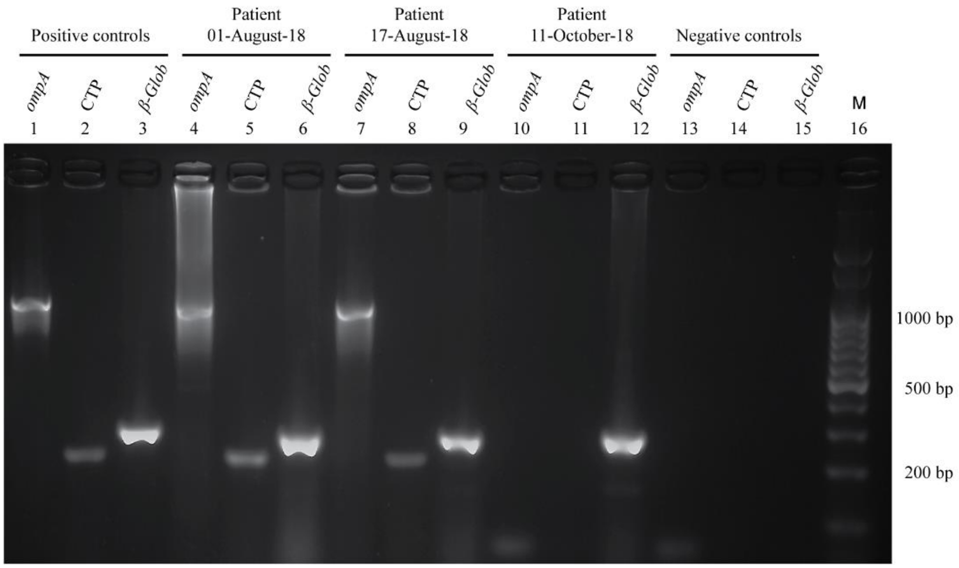

Figure S1: Electrophoretic analysis of the PCR amplification products of *C. trachomatis* and human β-globin genes in 2% agarose gels stained with GelRed Nucleic Acid Gel Stain (Biotium, San Francisco, USA). Lanes 1, 4, 7, 10 and 13: Heminested PCR of the *Chlamydia trachomatis*-specific *ompA* gene (amplicon: 990 bp). Lanes 2, 5, 8, 11 and 14: PCR of the *Chlamydia trachomatis*-specific cryptic plasmid (CTP) gene (amplicon: 210 bp). Lanes 3, 6, 9, 12 and 15: PCR of the human β-globin gene (amplicon: 230 bp; run as a control of DNA extraction and integrity and with the specific primers GH20/PC04). Lane 16: M, molecular weight marker (qLadder 100 bp precision, PB-L, Buenos Aires, Argentina). Lanes 1-2: DNA extracts from HeLa cells infected with *C. trachomatis* LGV-L2 strain 434/Bu were used as positive PCR controls for *ompA* and CTP. Lane 3: DNA extracts from semen sample was used as positive PCR control for human *β-globin* gene. Lanes13-15: DNAse/RNAse-free water samples were used as negative PCR controls for *ompA*, CTP and human *β-globin* genes, as indicated.

Figure S2. DNA sequences obtained for the strain detected in the patient's sample are shown. Alleles identified according to the MLST-Uppsala scheme and genovar identified based on *ompA* sequence analysis are indicated.

hctB – Allele 18 - 755 bp (5'-3')
CCCCCTCCCCCGGGGGATTTTAATACACCCCCCCCTTCTATGCTACGGAAGATTTTTTTGT
TGAAGCTACTCTTTTTGCTACAGTCTTCTTAGCTACAGCTTTTCTTACTGGAGCCTTTTTA
GCTGTTGCTTTCTTAGCTACAGGCTTACGAGCTGCTACAGTTTTTTTAGCTACTGTTTTGC
GAGCTACAGTCTTCTTAGCTGCAACTTTTCTTACTGGAGCCTTTTTAGCTGTTGCTTTCTT
AGCTACAGGCTTACGAGCTGCTACAGTTTTTTTAGCTACTGTTTTGCGGGCTACAGTCTT
CTTAGCTACAGCTTTTCTTACTGGAGCCTTTTTAGCTGTTGCTTTCTTAGCTACAGGCTTA
CGAGCTGCTACAGTTTTTTTAGCTACTGTTTTGCGGGCTACAGTCTTCTTAGCTGCAACTT
TTCTTACTGGAGCTTTTTTAGCTGCCGTCTTCTTAGCGGCAGGCTTACGTACTACAGTCTT
TCTGGCAGCAGTTTTTCTTGTGCTGCGTTTCTTTTGTACTCCCAACATGTTCATTCCCCTA
ATTAGACAGGTAACTACTACTTATTTGATCTATCGACAAGGAGAATGAAAAACTTTAAT
AAAAAATGGTTAGTTTTTAACTTTTTATTAAAAACTAACCACACATATTATTTAATATCG
CTAAACAATAACTATTTAGATAGAAATTTCTCCATATAATGAATTAAACAACGCACACC
AAATCCGTGCAAATTTTGGGGGGAAGGGG

CT058 – Allele 13 – 628 bp (5'-3')
AAGATAACTTTCTAGCCTCCGGACACTTGCATTCTTGCGCAAAACGGTGTGTTGTCCTAA
GCCCTTGGGCAAAAATTTTAAAAGTTAAGAGTTTATGTTTACATCGCTGTCCGCGATACA
GAATGCTATACGTCCTTCTTGTCAACTTCCTGTTTTGACTCCTAGACGCGCTCTCATTACT
TCTCTTGCGTCTGGAATCATTTTAGGACTTGCTGGTTGCGTGGTTGGCGTTTTAGCCTCCC
TTCCTGCCCTAATCGCCGTTTCTGCTGTTATTTTAGGTGTCAGTCTTTTTGCTTCAGGACT
ATTTCTCTGTCGATATGTTTGTCCCCCAAAAATTGTGTCCCGAAGACCTTCTACCGAACT
CCCTGCTGAACCTACTCCCGAGCTGCCTGAAATCAAAAGACCTAAACCTATAGCTCCTCC
TCCTCCAGATTTCATACCTCCAAAACCACTGAGAAGAACGATCGGTGAAATGCTTTTTGG
ATGGAACTGCATAGAATCGATAAGACAGATGCCGTTTTTCCTTGCTAATGACAAAACGC
CTCTGTTCTTCAGAAACCCTTCAGCAAGATTTAGAGCCTGGAATATTCCTTTCACTCATA
CTATTTTTGTCTCTCCTTCAGGCCAA

CT144 – Allele 19 – 462 bp (5'-3')
ATCTCTTTTGGCGGTCGTGTGAATTTGGCAGATAACACGATAACTTATAATCCGTATAAT
AATGGGGGAGGAGAAGTTGCGTTTACGGACATCAACTCTCGTCAAGGGAAGCAATACGT
TCCTTATGGGCTCTATAAGAATGGAGCGCCTAAAATAAGTATGCGTTCAGCGCTATCCG
GAGGCCACGTAGGATCTGGAGATACTAGAGGCTGGGGAGCAGAAGTGCTTTGGGATGC
CTATACTCAGCAGAGAGAGGATTTAGAAGATAAGGCTGTGACTTTTAGCCCTGCGAATA
GAGGTAAATTGTATTTTGAGGCATCTCCTGCGGCTCCCGTTTTGTTCCGTCTCTCCGTCTT
TATGAGAAAAAATGGAGACTGGCTAGATAATGGAGCAGGGGGACGTGTGATGCTCTAT
GTAAACACTAAAAAATTCTGCTGGGAAGACGATGCGACGCTTATTAGGA

CT172 – Allele 6 -383 bp (5'-3')
TTTGTTTTGGATATTTCCCTCAGTTATTCGAAAAACAAAAAAATCTATTAGAAGATCTTA
TTTACAATAAACTGTTTGTAGAAAAAACCGTAGTAATGGGTGAGGGAGCATGTGTTGTC
AATCTAGCAATAGGGGGGGGGTAGACTATTTTACTTTAAAACCTCTACCGAATTTGGTTG
AGAAAAATGAATTATCACAACACTGTTTGTAAAAACCAGCATGTTTTTCTTGGCAAAAA
GACTAGTTCAGTTAAATAAAAATCCTTTCTTACTCAAAAAGTTTTCAGAAACAACGGTTC

TTTTTATATTCGAACGACAACTTAAAATGTGGGAAGGTTATTCTATAGACGAGAATAATT
ATATATCTGATTATAACAGGGAATT

pbpB – Allele 28 – 605 bp (5’-3’)
TGTTGCTGTTGTGCAGCATGAGAAAAAGAAGGAAGAAGCTTATCGTCCTCAGCGTAGAA
GTGTTCCTGAGCATTGTGATCGCGCAGGAGTATGTGATCGTTTTGGTAAAACGCTGGCA
GAAAACGTTTTACAATATAATGTGGGGATCTCTTATCGAGCGATACGTGATATTCCGAC
GCGTGTTTGGCATACAGATGAGCAAGGGAATAAGAGGCTTGTTCCTGTTCGTAAGGATT
ACATTAAAAAATTTGCAGATTTTTTGGCTCAAGAGCTGCATATGGACCGTGATTTCGTTG
AAGATACGATCCATGCCAAAGCTTCTGTATTAGGGTCTGTACCTTACATCCTTCAGACTA
ATGTATCTGAGCGTACTTTTTTAAGGCTCAAGATGCTGGAGAAGGATTGGCCAGGATTG
CATGTCGAATCTTCGGTTCGCAGACATTATCCTGAAGGGCGCACAGTAGCTGATTTGTTG
GGGTATGTCGGACCTATTAGCGCAGAAGAACATAGAAAAATTACGAGGGAGTTAGGAA
ACCTAAGAGAATGCATTCGTGCTTATGAAGAGGGTGAGGATCCTAAATTTCCTGCAGGA
ATATCGAGCGTCG

ompA - Genovar L2 - 967 bp (5’-3’)
TCGACGGAATTCTATGGGAAGGTTTCGGCGGAGATCCTTGCGATCCTTGCACCACTTGGT
GTGACGCTATCAGCATGCGTATGGGTTACTATGGTGACTTTGTTTTCGACCGTGTTTTGC
AAACAGATGTGAATAAAGAATTCCAAATGGGTGCCAAGCCTACAACTGCTACAGGCAAT
GCTGCAGCTCCATCCACTTGTACAGCAAGAGAGAATCCTGCTTACGGCCGACATATGCA
GGATGCTGAGATGTTTACAAATGCTGCTTACATGGCATTGAATATTTGGGATCGTTTTGA
TGTATTCTGTACATTAGGAGCCACCAGTGGATATCTTAAAGGAAATTCAGCATCTTTCAA
CTTAGTTGGGTTATTCGGAGATAATGAGAACCATGCTACAGTTTCAGATAGTAAGCTTGT
ACCAAATATGAGCTTAGATCAATCTGTTGTTGAGTTGTATACAGATACTACTTTTGCTTG
GAGTGCTGGAGCTCGTGCAGCTTTGTGGGAATGTGGATGCGCGACTTTAGGCGCTTCTTT
CCAATACGCTCAATCCAAGCCTAAAGTCGAAGAATTAAACGTTCTCTGTAACGCAGCTG
AGTTTACTATCAATAAGCCTAAAGGATATGTAGGGCAAGAATTCCCTCTTGATCTTAAA
GCAGGAACAGATGGTGTGACAGGAACTAAGGATGCCTCTATTGATTACCATGAATGGCA
AGCAAGTTTAGCTCTCTCTTACAGACTGAATATGTTCACTCCCTACATTGGAGTTAAATG
GTCTCGAGCAAGTTTTGATGCAGACACGATTCGTATTGCTCAGCCGAAGTCAGCTACAA
CTGTCTTTGATGTTACCACTCTGAACCCAACTATTGCTGGAGCTGGCGATGTGAAAGCTA
GCGCAGAGGGTCAGCTCGGAGATACCATGCAAATCGTTTCCTTGCAATTGAACAAGATG
AAATCTAGAAAATC

Table S1. Specific primers used for uropathogens detection

Pathogen	Gene	Primer	Oligonucleotides (5′– 3′)	Amplicon size (bp)	Reference
Chlamydia trachomatis	*ompA*	SEROA1	ATGAAAAAACTCTTGAAATCGG	990	Lan *et al.*, (1995)
		SEROA2	TTTCTAGAYTTCATYTTGTT		
		PCTM3	TCCTTGCAAGCTCTGCCTGTGGGGAATCCT		
	CTP	CTP1	TAGTAACTGCCACTTCATCA	201	
		CTP2	TTCCCCTTGTAATTCGTTGC		
Ureaplasma urealyticum	urease	U4	ACGACGTCCATAAGCAACT	429	Diaz *et al.*, (2010)
		U5	CAATCTGCTCGTGAAGTATTAC		
Mycoplasma hominis	16S rRNA	RNAH1	CAATGGCTAATGCCGGATACGC	334	
		RNAH2	GGTACCGTCAGTCTGCAAT		
Mycoplasma genitalium	MgPa	MG-1F	ACCTTGATGGTCAGCAAAACTT	193	Gimenes *et al.*, (2014)
		MG-2R	CCTTTGATCTCATTCCAATCAGTA		
Herpes Simpex Virus type 1	Glycoprotein G (US4)	HSV1-1F	CTGTGGTGTTTTTGGCATCA	123	
		HSV1-2R	GGTTGTGGAGGAGACGTTG		
Herpes Simpex Virus type 2	Glycoprotein D (US6)	HSV2-1F	CATGGGGCGTTTGACCTC	249	
		HSV2-2R	TACACAGTGATCGGGATGCT		
Human Papilloma Virus	L1	MY09	CGTCCMAARGGAWACTGATC	450	
		MY11	GCMCAGGGWCATAAYAATGG		
Trichomonas vaginalis	Adhesive protein	TV-1F	CCAGAAGTGGGCTACACACC	170	
		TV-2R	ATACCAAGGCCGGAAGCAC		
Treponema pallidum	47-kDa membrane immunogen	TP-1F	GGAGAAGTTTCACTTCGTGGA	291	
		TP-1R	CTCGCGTCATCACCGTAGTA		
Neisseria gonorrhoeae	PorA pseudogene	NG-1F	CGGCAGCATTCAATTTGTT	162	
		NG-2R	AAAAAGCCGCCATTTTTGTA		

Table S2. *Chlamydia trachomatis* MLST (Uppsala) allelic profile

Locus	Identity (%)	Coverage (%)	Alignment length	Allele length	Gaps	Allele
hctB	100	100	755	686	0	18
CT058	100	100	628	600	0	13
CT144	100	100	462	448	0	19
CT172	100	100	383	362	0	6
pbpB	100	100	605	602	0	28
				**Sequence type:**	141	

Gene loci were analyzed according to the MLST Uppsala scheme. Identity and coverage (expressed as percentage), alignment and allele lengths and gaps of each loci's DNA sequence obtained (number of base pairs) with respect to the reference sequence retrieved by the MLST-Uppsala database (https://pubmlst.org/) are shown. The number of the allele identified is also indicated. The sequence type (ST) obtained for the strain detected in the patient's sample exactly matched with *C. trachomatis* ST141.

References

Diaz, N., Dessi, D., Dessole, S., Fiori, P.L., and Rappelli, P. (2010). Rapid detection of coinfections by Trichomonas vaginalis, Mycoplasma hominis, and Ureaplasma urealyticum by a new multiplex polymerase chain reaction. Diagn Microbiol Infect Dis 67(1), 30-36. doi: 10.1016/j.diagmicrobio.2009.12.022.

Gimenes, F., Medina, F.S., Abreu, A.L., Irie, M.M., Esquicati, I.B., Malagutti, N., *et al.* (2014). Sensitive simultaneous detection of seven sexually transmitted agents in semen by multiplex-PCR and of HPV by single PCR. PLoS One 9(6), e98862. doi: 10.1371/journal.pone.0098862.

Lan, J., Melgers, I., Meijer, C.J., Walboomers, J.M., Roosendaal, R., Burger, C., *et al.* (1995). Prevalence and serovar distribution of asymptomatic cervical Chlamydia trachomatis infections as determined by highly sensitive PCR. J Clin Microbiol 33(12), 3194-3197. doi: 10.1128/jcm.33.12.3194-3197.1995.
